# Points in Two English and One Scotch Hospital Reports

**Published:** 1907-06-15

**Authors:** 


### POINTS IN TWO ENGLISH AND ONE SCOTCH HOSPITAL REPORTS.

### The Cumberland Infirmary.

It may be interesting to call attention to this institution, which is one of the smaller county hospitals, where the average daily number of occupied beds is 87.5. The report shows how great has been the advance in the intelligence brought to bear upon hospital literature, and some of the illustrations are better reproduced than any we have yet seen in a report of this kind. They point to evident care in the internal management, and display evidence of an endeavour to make this hospital up to date and attractive. The tables contained in the report are valuable, because they show the working of the institution in detail for ten consecutive years. There is evidence that the management of the institution has progressed since we inspected it some fifteen years ago. It is instructive to note that, whereas the average of the daily occupied beds in 1906 was one more than ten years ago, the number of in-patients under treatment had increased from 897 to 1,127. These figures indicate an advance in hospital methods, and it is creditable to the management that the cost of maintenance, despite the increase in the actual number of in-patients, has been kept well in hand. The number of annual subscriptions has steadily increased of late, and the present income, including legacies, was, roughly, £2,200 greater in 1906 than in 1897.

### General Hospital, Birmingham.

We had the pleasure of inspecting this institution recently, and were pleased to find everything in excellent order, the wards beautifully kept, and the condition of the whole institution, from the administrative point of view, testified to the excellence of the management in all departments. We soon contracted a bad headache, however, owing to the continuous air pressure and atmospheric conditions, due to the system of ventilation, which we hold to be quite unsuitable to a temperate climate like England. It is instructive to note that in hospitals more recently built under the Plenum system that system has been excluded from the buildings occupied by the resident officers and the nursing staff. We are glad to record that an attempt is being made to sift the out-patients at this hospital, with the object of securing the undivided attention of the visiting staff for cases that are really serious. Successful efforts have been made to secure the dedication of a large number of beds, by a single payment of £1,250 per bed, and the list of the donors of such beds now occupies two pages of the report. The growth in operative surgery is emphasised by the fact that the number of house surgeons has been increased from five to seven, three thousand three hundred and forty-four operations having been performed in 1906. The revenue shows a tendency to halt, if not to decrease, but careful management has resulted in an increase of the invested funds. The average cost of each in-patient is £3 6s. 10d.; the daily average of in-patients was 304; and the cost per occupied bed nearly £78, the last item showing a reduction on the four previous years. The report is illustrated by photographs, but some of the blocks are either defective, or the printing leaves much to be desired.

### Glasgow Royal Infirmary.

The report of the Glasgow Royal Infirmary is so much of a business document, and so issued, that it probably fails to interest ordinary people in the way that hospital reports, prepared on modern lines, undoubtedly do. The present form of the report is one which has been followed for a great number of years, and it seems to us that the time is approaching, if it has not already arrived, when the managers should seek the co-operation of some governors with literary ability and ideas, and constitute them a committee to re-cast the report, modernise it, and make it as attractive as possible. If this were done it should have a marked influence in producing funds for the support of the institution. The most remarkable feature about the report is the evidence it contains that the artisan classes are the backbone of the voluntary revenue, for their contributions have continued to extend through the workshops and warehouses in increasing volume each year. Previous to 1852 the artisans gave less than £1,000 a year to the Royal Infirmary, but during 1906 they paid in no less than £8,816. On the other hand, the annual subscriptions show a remarkable falling off in 1906 compared with 1905. Of upwards of seventeen thousand subscribers of under £1 a year each, nearly six thousand withdrew their subscriptions in 1906, and their example was followed by one hundred and seventy subscribers of larger amounts varying from £100 to £1 per annum. No explanation of these figures is contained in the report, but the figures are remarkable, and we should like to have an explanation of their meaning, and of the causes which have led to a falling off of £818 in annual subscriptions in one year, together with the disappearance of so many subscribers from the books of this important hospital. Another remarkable point is that the ordinary expenditure shows an increase in every item except printing, the total increase amounting to £2,605 in 1906 compared with 1905. This is the more unaccountable, seeing that the number of admissions and of patients under treatment show a decrease in 1906 compared with the previous year.

